# 

**Published:** 1887-10

**Authors:** 


					﻿yolume LV.|
OCTOBER, 1887.
[Number 4
The CHICAGO
Medical
Journal
EXAMINER
EDITORScg hs agasa ghcgs ac
(LARK & LONGLEY CHICAGO'
i ' v Puri	I
hniiiiiiniiiiiHiwiii.iu.... A...YXD_1^1>^rA5TA\??uiHuuiiiUilUU<^h I
Entered at the Post Office at Chicago, for transmission through the mails as second-class matter.
				

